# Aquaporin‐Incorporated Graphene‐Oxide Membrane for Pressurized Desalination with Superior Integrity Enabled by Molecular Recognition

**DOI:** 10.1002/advs.202101882

**Published:** 2021-08-16

**Authors:** Chang Seon Lee, Insu Kim, Jae Won Jang, Dae sung Yoon, Yun Jung Lee

**Affiliations:** ^1^ Department of Energy Engineering Hanyang University Seoul 04763 Republic of Korea; ^2^ School of Biomedical Engineering Korea University Seoul 02841 Republic of Korea

**Keywords:** aquaporin, biomimetic, graphene oxide, membranes, molecular recognition

## Abstract

Aquaporins (AQPs), the natural water channel, have been actively investigated for overcoming the limitations of conventional desalination membranes. An AQP‐based biomimetic high‐pressure desalination membrane is designed by tethering AQP‐carrying red blood cell membrane (RBCM) vesicles onto graphene oxide (GO). RBCMs with AQPs are incorporated into GO based on the molecular recognition between the integrin of RBCM and Arginine‐Glycine‐Aspartate (RGD) ligand on the GO surface. GO is pre‐functionalized with the Glycine‐Arginine‐Glycine‐Aspartate‐Serine peptide to capture RBCMs. RBCMs are inserted between GO flakes through the material‐specific interaction between integrin of RBCM and RGD ligand, thus ensuring sufficient coverage of channels/defects in the GO for the full functioning of the AQPs. The incorporated AQPs are not completely fixed at the GO, as tethering is mediated by the integrin–RGD pair, and suitable AQP flexibility for appropriate functioning is guaranteed without frictional hindrance from the solid substrate. The integrity of the GO–RBCMs binding can provide mechanical strength for enduring high‐pressure reverse‐osmosis conditions for treating large amounts of water. This biomimetic membrane exhibits 99.1% NaCl rejection and a water permeance of 7.83 L m^−2^ h^−1^ bar^−1^ at 8 bar with a 1000‐ppm NaCl feed solution, which surpasses the upper‐bound line of current state‐of‐the‐art membranes.

## Graphical abstract

Aquaporin (AQP)‐based biomimetic desalination membranes are designed by tethering red blood cell membranes (RBCMs), the aquaporin carrying vesicles, on graphene oxide (GO) through the material‐specific affinity between the integrin of the RBCM and Glycine‐Arginine‐Glycine‐Aspartate‐Serine (GRGDS) peptides pre‐functionalized on GO. The resulting GO‐GRGDS‐AQP membrane demonstrates high water permeance and NaCl rejection above 99% with sufficient mechanical integrity for enduring pressurized desalination.

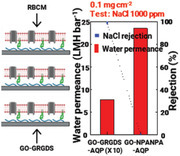

## 1 Introduction

Water contamination and clean‐water shortages have become major problems in daily life.^[^ ^1^, ^2^ ^]^ Water purification using a membrane plays a significant role in addressing these problems because of the high efficiency and eco‐friendly nature of the membrane‐based process.^[^ ^3^, ^4^ ^]^ Water‐purification membranes, which allow the transfer of water molecules while simultaneously rejecting undesirable ions/solutes in a highly pressurized system, can be classified into two types, namely reverse osmosis (RO) membranes and nanofiltration (NF) membranes. Polymeric membranes such as thin‐film composite (TFC) polyamide (PA) membranes have been considered as the standard for RO and NF processes over the last four decades.^[^ ^5^ ^]^ Numerous studies have been conducted to improve the water permeance of polymeric membranes while maintaining their selectivity at a certain level.^[^ ^6^, ^7^, ^8^, ^9^, ^10^ ^]^ However, an increase in water permeance of the membranes generally results in lower selectivity for water molecules. Replicating the features of natural water channels has been regarded as an unconventional and radically novel approach for overcoming this selectivity–permeance trade‐off behavior in polymeric membranes.

Aquaporins (AQPs) are biological water channels that exhibit high water permeability and high rejection of other molecules and salt (or ions).^[^ ^11^, ^12^, ^13^ ^]^ Since the finding of water molecule transport mediated by AQPs in human body,^[^ ^14^ ^]^ AQPs have been actively employed in the fabrication of biomimetic membranes for water treatment. After the first AQP incorporated biomimetic membrane had shown a two‐order increase in water permeability than that of the commercial polymeric membranes,^[^ ^15^ ^]^ various studies have been conducted in attempts to emulate novel features of AQPs in artificial membranes.^[^ ^16^ ^]^ Attaching AQP‐mimetic peptides on pillar[5]arene^[^ ^17^ ^]^ and graphene oxide^[^ ^18^ ^]^ membranes also significantly increased their water permeance; however, they were still unable to reject ionic species. Instead of imitating AQPs by tethering AQP‐mimetic peptides onto polymeric membranes, the direct incorporation of natural AQPs into the membrane has been proposed. The porous polycarbonate track etched membrane incorporated with AQP proteoliposomes exhibited an NaCl rejection of ≈97.8%.^[^ ^19^ ^]^ Although the ion‐rejection capability of AQPs was successfully transferred to polymeric membranes, the inherent problem such as defect made the ion‐rejection performance of biomimetic membranes subpar.^[^ ^20^ ^]^ Also, the water permeability of this membrane was lower than that of commercial RO membranes because the transport properties were measured in forward osmosis (FO) systems. Because the integrity of the membrane was insufficient for withstanding a high‐pressure RO system, the measurement was conducted in an FO system under a relatively low pressure as compared to that of the RO system. In this regard, the mechanical strength of the membrane to withstand high‐pressure conditions is important for achieving a high flux and salt rejection capability. In addition, streamlining the fabrication process is another challenge for producing large‐area membranes because the majority of biomimetic membranes usually require intricate techniques for their fabrication, which limits the scaling up of the water treatment process.

**Figure 1:**
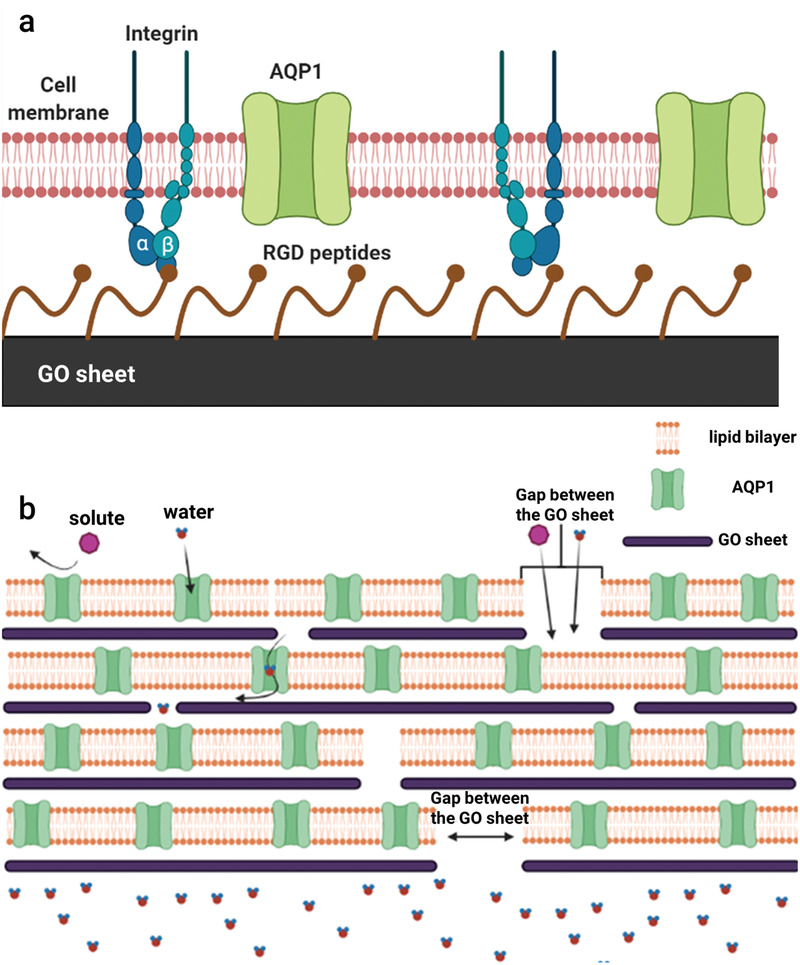
a) Schematic of RBCM binding onto GO‐GRGDS via integrin‐RGD ligand molecular recognition. b) Schematic of mass transport procedure through GO‐GRGDS‐AQP membrane.

Herein, we report an AQP‐incorporated water‐treatment membrane with superior integrity under high‐pressure operation based on the principle of molecular recognition: Arginine‐Glycine‐Aspartate (RGD)–integrin binding. The ‐RGD‐ sequence is known as a cell‐binding peptide sequence, and the engineered vesicles incorporating the AQP proteins can have integrins that selectively bind to specific RGD peptides in the extracellular matrix.^[^ ^21^, ^22^ ^]^ ‐RGD‐ peptides tethered on the substrate can bind with the integrin of the vesicles, boost the AQP loading to the substrate, and enhance mechanical durability under high‐pressure RO conditions. In this study, graphene oxide (GO) membranes were adopted as a substrate membrane because oxygen‐containing functional groups on the GO surface can be easily modified with the desired peptides.^[^ ^23^, ^24^ ^]^ Moreover, GO itself can be used as an appropriate water‐purifying membrane^[^ ^25^, ^26^ ^]^ through size sieving because the interlayer galleries between stacked graphene flakes serve as molecular pathways.^[^ ^27^ ^]^ However, the spacing between GO sheet swells when immersed in water which result in significant decrease in ion selectivity.^[^ ^25^ ^]^ Although this swelling issue for GO laminates limits its use for desalination, it also means that GO can offer a space for functionalizing molecules such as peptides or bilayers.^[^ ^18^ ^]^ Accordingly, we used GO laminates as solid‐state scaffold for biomimetic membrane. We used GO films functionalized with ‐RGD‐peptides on a mixed cellulose ester (MCE) layer as the substrate for incorporating the AQPs, and the MCE was used to ensure mechanical strength because the thin free‐standing GO films are generally damaged in high‐pressure systems. A schematic description of the tethering of AQPs onto the GO through integrin–RGD molecular recognition is presented in **Figure** **1**a. We expected that the lipid bilayer of the vesicle and basal plane of the GO membrane would work as a barrier for the transport of both water and salt ions, and the red blood cell membranes (RBCMs) inserted between the GO layers effectively passivate the defects in GO such that only AQPs selectively transport water, thus achieving a high water flux in the high‐pressure RO system and good salt‐rejection capability. As AQP‐carrying vesicles, red‐blood cells (RBCs) containing abundant AQPs in their lipid bilayer were selected and engineered. Especially, AQP1, which shows highest single‐channel permeability among various AQPs, is included in RBCs.^[^ ^20^ ^]^ The RBCs obtained after the removal of their intracellular components are called RBC membranes, which serve as vesicles for the AQP1 (AQP, hereafter). RBCs are reported to possess an integrin *α*_IIb_ *β*_3_, called the fibrinogen receptor, in the lipid bilayer, and this integrin selectively binds to the RGD domain.^[^ ^21^, ^28^, ^29^ ^]^ GRGDS, the RGD sequence of fibrinogen, was selected as a cell‐binding short peptide and functionalized on the surface of GO. The GRGDS‐functionalized GO (GO–GRGDS) membrane successfully captures RBCMs with AQPs and becomes a mechanically stable GO‐based membrane functionalized with AQPs (GO–GRGDS–AQP) through the binding between the integrin and RGD sequences based on molecular recognition. For the incorporation of the RBCM vesicles onto the planar GO surface, circular vesicles were flattened by external forces such as sonication as the vesicles interacted with the GO. As the AQPs were incorporated into the GO membrane as they were being embedded in the RBCM vesicles and not in direct contact with the GO substrate but mediated by the RGD peptide, we expect that the function of the AQPs could be retained in GO membranes with proper movement of the AQPs. Complete fixation within the rigid matrix or frictional hindrance from the substrate are known to limit the full functioning of AQPs.^[^ ^30^ ^]^ In addition to these functional limitations, complicated processes and high costs are some of the greatest hurdles in the practical use of biomimetic membranes for water purification.^[^ ^31^ ^]^ The mass transfer procedure through the GO‐GRGDS‐AQP membrane is schematically presented in Figure 1b. The mass transfer occurs through the hole between GO flakes, interlayer galleries between GO sheets, and AQP1 in bilayer. Water molecules permeate through AQP1 while unwanted solutes/ions are rejected by AQP1. Biomimetic membranes that exhibit both high water permeance and salt rejection generally have small effective areas as compared to commercial water‐purification membranes because of the complexity of their fabrication process.^[^ ^17^, ^32^, ^33^ ^]^ In this study, we developed a simple and scalable fabrication process for GO–GRGDS–AQP to obtain, not only a higher water permeance (LMH bar^−1^), but a higher water flux (LMH) than those of the commercial membranes for realizing cost‐efficient biomimetic water treatment.

## 2 Results and Discussion

Our fabrication process for the GO–AQP membrane starts with GO functionalization. The functionalization process is schematically presented in Figure S1, Supporting Information. A pristine GO solution was pre‐treated with carboxylated GO (GO–COOH) to enhance the carbodiimide coupling (1‐ethyl‐3‐(3‐dimethlyaminopropyl)‐carbodiimide (EDC)/ *N*‐hydroxysuccinimide (NHS) coupling) reaction between GO and the GRGDS peptides. Oxygenated surface functional groups on GO, such as hydroxyl (‐OH) and epoxide (‐O‐) groups, turned into carboxyl (‐COOH) groups during this pretreatment. GRGDS, the RGD motif of fibrinogen, was then anchored to the GO–COOH surface through EDC/NHS coupling,^[^ ^34^ ^]^ thus resulting in GO–GRGDS. The functionalization of GO with GRGDS was examined via X‐ray photoelectron spectroscopy (XPS). The high‐resolution C1s spectrum of GO–COOH in Figure S2a, Supporting Information shows that carboxyl groups are the dominant functional groups of GO–COOH. Peptide‐functionalized GO (GO–GRGDS) has a similar C1s profile but a relatively higher intensity of ‐COOH (Figure S2b, Supporting Information) because of the presence of additional carboxylic groups in the peptides. Successful functionalization of GRGDS on GO was confirmed through a comparison of the high‐resolution N1s spectra of GO–COOH and GO–GRGDS. The presence of nitrogen in the form of an amide in GO–GRGDS was verified, while GO–COOH exhibited no noticeable amide bonds (Figure S2c, Supporting Information).

To coat the RBCM onto the GO–GRGDS, the RBCM was sonicated, and the resulting individual RBCM vesicles exhibited a mean diameter of 169 ± 4 nm (Figure S3, Supporting Information). These RBCM vesicles were mixed with the GO–GRGDS solution and then sonicated.^[^ ^35^ ^]^ During sonication, the RBCM vesicles were broken and reconstituted on the surface of the GO–GRGDS in the form of a lipid bilayer.^[^ ^36^ ^]^ The resulting GO with attached RBCMs was still well‐dispersed and named as the GO–GRGDS–AQP solution. The GO‐based water‐treatment membrane was fabricated by vacuum‐filtrating GO‐based solutions through the MCE support with a total mass loading of 0.1 mg cm^−2^. The thickness of the GO–GRGDS membrane with a total mass loading of 0.1 mg cm^−2^ was found to be 314 nm via scanning electron microscopy (SEM) as presented in Figure S4a, Supporting Information. GO‐based membranes with AQPs were produced as a composite layer of the underlying GO–GRGDS layer and GO–GRGDS–AQP surface layer. The ratio of GO–GRGDS:GO–GRGDS–AQP was set as 8:2 (0.08 mg cm^−2^ GO–GRGDS and 0.02 mg cm^−2^ GO–GRGDS–AQP). The total thickness of the GO–GRGDS–AQP membrane is approximately 611 nm (Figure S4b, Supporting Information).

**Figure 2:**
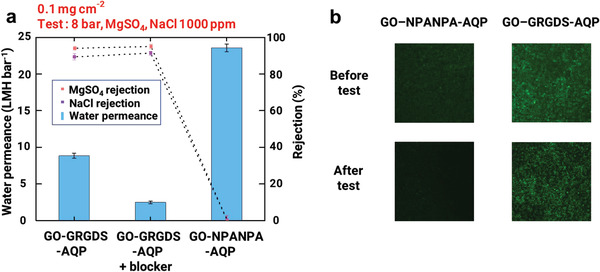
AQP‐incorporated GO‐based membranes. a) Water permeance and rejection of GO–GRGDS AQP, GO–GRGDS–AQP +AQP blocker, and GO–NPANPA–AQP at 8 bar with the same mass loading (0.1 mg cm^−2^). Feed solution was 1000‐ppm aqueous solution of MgSO_4_ and NaCl. b) Fluorescence images of GO–GRGDS–AQP and GO–NPANPA–AQP before and after permeation test.

The permeate flux and rejection rate of NaCl and MgSO_4_ solutions through the GO–AQP membrane were evaluated in a pressure‐driven dead‐end filtration cell (stirred cell). Hexapeptide sequenced asparagine‐proline‐alanine‐asparagine‐proline‐alanine (NPANPA) was selected as a control as NPA is a constituent motif of AQP, and the length of NPANPA is similar to that of GRGDS. The resulting control sample is denoted as GO–NPANPA–AQP. A GO–NPANPA–AQP membrane was formed with underlying 0.08 mg cm^−2^ GO–NPANPA and a 0.02 mg cm^−2^ surface GO–NPANPA–AQP layer. In addition, an AQP blocker was added to the GO–GRGDS–AQP membrane (denoted as GO–GRGDS–AQP +AQP blocker) for the purpose of comparison to verify the water permeation through the AQPs of the GO–GRGDS–AQP membrane. AQP blockers inhibit the passage of water molecules through AQPs by blocking the narrowest part of the water channel^[^ ^37^ ^]^; thus, a comparison of the water flux with and without an AQP blocker could confirm whether the water permeation occurs primarily through the AQPs or defects in GO. The water permeance and rejection rate for the aqueous solution of 1000 ppm NaCl and MgSO_4_ through various GO‐based membranes having the same mass loading (0.1 mg cm^−2^) at an applied pressure of 8 bar are presented in **Figure** **2**a. The water permeance through the GO–NPANPA–AQP membrane was 23.56 L m^−2^ h^−1^ bar^−1^ (LMH bar^−1^), which is almost 2.5 times greater than that through GO–AQP membranes (8.84 LMH bar^−1^). However, the GO–NPANPA–AQP membrane showed virtually no rejection of NaCl and MgSO_4_, while the GO–GRGDS–AQP membrane exhibited 94% rejection of MgSO_4_ and 89% rejection of NaCl. This dramatic difference in the ion‐rejection capability between the two membranes is likely due to the difference in the binding affinity of RBCM (with embedded AQPs) to the peptide motif on the GO surface. As there is no specific affinity between NPANPA peptides and RBCMs, the interaction between these two must be a weak random interaction. Such weak attachment is fragile under applied pressure, and the RBCM could be washed away during the test under pressure. Thus, the water permeation occurs primarily through the interlayer space and defects in GO while exhibiting a higher permeance but no salt‐rejection capability. In contrast, a strong integrin–RGD ligand binding between RBCM and GRGDS would maintain the superior integrity of the RBCM (and AQPs embedded in RBCM) attachment to GO under the applied pressure. Thus, GO–GRGDS–AQP exhibits salt‐rejection properties endowed by AQPs in the RBCM. The addition of an AQP blocker to the GO–GRGDS–AQP membrane (GO–GRGDS–AQP + AQP blocker) resulted in a significant decrease in water permeance (2.9 LMH bar^−1^) to 1/3 that of the pristine GO–GRGDS–AQP, which represents the theoretical decrease in water permeance due to the blocker attachment to the AQP.^[^ ^37^ ^]^

To visually verify the successful functionalization of the RBCM on GO, RBCMs on GO were imaged using fluorescence confocal microscopy before and after the water purification test. The fluorescence marker was labeled to the phosphate head group in the lipid bilayer of the RBCM such that we could trace the presence of the RBCMs on GO. The fluorescence images of each membrane are presented in Figure 2b. The fluorescence‐marked GO–GRGDS–AQP membrane before the test exhibited a relatively uniform and strong fluorescence on the GO membrane. After the test, the fluorescence intensity of GO–GRGDS–AQP was slightly reduced but still uniform, and considerable fluorescence was detected, thus indicating that the RBCM vesicles were stably anchored on the GO surface even after the pressurized transport test. In contrast, the labeled GO–NPANPA–AQP membrane demonstrated weak and non‐uniform fluorescence even before the test. Moreover, the fluorescence intensity of the GO–NPANPA–AQP membrane significantly decreased and almost disappeared after the permeation test. This confirms that the RBCM vesicles were weakly bound to GO–NPANPA and were washed away during the fabrication process and pressurized test, which is consistent with the result of the permeation test presented in Figure 2a.

**Figure 3:**
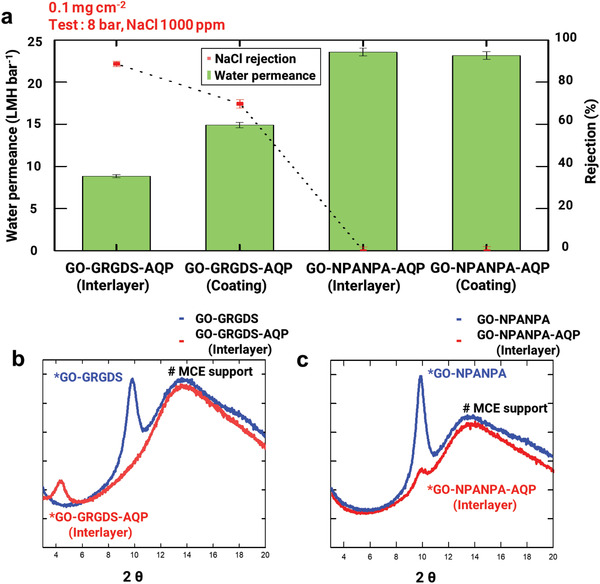
Comparison of RBCM vesicle incorporation methods into GO membrane: Interlayer versus vesicle fusion coating. a) Water permeance and rejection of pristine GO–GRGDS–AQP (interlayer), GO–GRGDS–AQP (vesicle fusion coating), GO–NPANPA–AQP (interlayer), and GO–NPANPA–AQP (vesicle fusion coating) at 8 bar with same mass loading. Feed solution was aqueous NaCl of 1000 ppm. XRD of b) GO–GRGDS and GO–GRGDS–AQP (interlayer), c) GO–NPANPA and GO–NPANPA–AQP (interlayer).

In nano‐biotechnology, one of the common methods of incorporating vesicles into the solid substrate is vesicle fusion, wherein the vesicles are coated onto the pre‐formed substrate.^[^ ^38^ ^]^ In our study, we introduced a different approach for incorporating AQPs into a GO membrane. The RBCM vesicles with embedded AQPs were mixed with the GO solution and stacked with the GO layers. Here, the AQP‐incorporated GO membranes fabricated using this method are denoted as interlayer membranes (GO–GRGDS–AQP (interlayer) and GO–NPANPA–AQP (interlayer)). To compare the efficiency of the loading of the RBCMs into GO membranes, GO‐peptide‐AQP membranes were also fabricated using the vesicle fusion method (GO–GRGDS–AQP (vesicle fusion coating) and GO–NPANPA–AQP (vesicle fusion coating)). The RBCM vesicles were placed on a premade GO–GRGDS membrane and incubated for 1 h at 50 °C.^[^ ^38^ ^]^ The unbound RBCM vesicles were washed with phosphate‐buffered saline (PBS). In a previous study, we analyzed the thickness of the RBCM per unit area according to the amount of RBCM.^[^ ^39^ ^]^ In the case of the vesicle fusion method, the RBCM membrane was designed to have a thickness of approximately 400 nm to achieve sufficient mechanical strength, which is equivalent to 80 cell membranes. Therefore, the total thickness of the GO–GRGDS–AQP (vesicle fusion coating) membrane was estimated to be ≈700 nm, while that of the GO–GRGDS–AQP (interlayer) was 611 nm, as shown in Figure S4b, Supporting Information. In this vesicle fusion method, the integrin of the RBCM vesicles may interact only with the GRGDS peptides exposed at the top surface of the GO–GRGDS membrane. The different RBCMs incorporation into the GO membranes via the vesicle fusion and interlayer method are schematically depicted in Figure S5, Supporting Information. The transport properties of the fabricated membranes were examined using a 1000‐ppm NaCl aqueous solution at an applied pressure of 8 bar (**Figure** **3**a). The control GO–NPANPA membrane was also fabricated and tested under the same conditions. The GO–GRGDS–AQP (vesicle fusion coating) membrane exhibited a much higher water permeance (14.92 LMH bar^−1^) than GO–GRGDS–AQP (interlayer) (8.84 LMH bar^−1^), but with a relatively low rejection rate of 70%, despite the higher total membrane thickness. This low salt rejection and high water permeance of the GO–GRGDS–AQP (vesicle fusion coating) membrane could be an indicator of the insufficient passivation of defects in GO by the RBCMs owing to vesicle incorporation by surface fusion. Vesicle incorporation onto the solid substrate by the vesicle fusion method was reported to be efficient when the surface of the substrate is fairly flat; however, the GO membrane fabricated using vacuum filtration exhibits a surface root mean square roughness (Rq) of 111 nm (Figure S6, Supporting Information). The RBCM layers on the solid surface could not perfectly cover the rough surface, thus exposing the edges and defects of the stacked GO layers. In contrast, the GO–NPANPA–AQP membranes did not exhibit apparent differences in either permeance or rejection: both membranes produced using vesicle fusion and the interlayer method exhibited high water permeance and no salt‐rejection properties. This result is in agreement with the insufficient incorporation of the RBCMs into GO–NPANPA‐based membranes via either interlayer or vesicle fusion methods because of the weak or non‐specific interaction between the RBCMs and NPANPA.

To gauge the internal structure of the GO‐based membranes, the interlayer spacing between the GO layers was estimated using X‐ray diffraction (XRD). The diffraction peaks of each membrane were positioned at 2*θ* = 4.12 (GO–GRGDS–AQP (interlayer)), 9.87 (GO–GRGDS), 9.91 (GO–NPANPA–AQP (interlayer)), and 9.90 (GO–NPANPA) (Figure 3b,c). The corresponding interplanar distance calculated using Bragg's law (n*λ* = 2d sin*θ*) is 21.36 Å and 9.66 Å for GO–GRGDS–AQP (interlayer) and GO–GRGDS, respectively. Thus, the GO–GRGDS–AQP (interlayer) exhibited a channel size almost 2.5 times larger than that of GO–GRGDS. For the non‐specific NPANPA control samples, both GO–NPANPA and GO–NPANPA–AQP (interlayer) presented an interlayer spacing of 9.58 Å, which is similar to GO–GRGDS with no difference before and after the RBCM coating. These results indicate that when GO–AQP membranes are fabricated using the interlayer method, the RBCM vesicles are tethered on the surface of each GO–GRGDS layer and inserted between the GO layers, thus enlarging the interlayer space of GO‐GRGDS, while the RBCMs were not incorporated between the GO layers of GO–NPANPA owing to the weak and non‐specific binding. Also, the interplanar distance measured with the XRD data is in line with the previous observation of the thickness of RBCM. The thickness of RBCM in completely dried state observed with TEM was ≈2 nm,^[^ ^40^ ^]^ which is similar to our calculated interplanar distance, 2.13 nm. The analyses presented in Figure 3 support our rationale for the deployment of molecular recognition chemistry (RGD ligand‐integrin interaction) for the fabrication of mechanically stable, nanoscale incorporation of AQPs into solid‐state artificial membranes.

As GO–GRGDS–AQP membranes fabricated using the interlayer method demonstrated acceptable levels of salt rejection with sufficient water permeance, all the GO‐based membranes were fabricated using the interlayer method hereafter. The GO‐based membranes with AQPs comprised an underlying GO–GRGDS layer and a GO–GRGDS–AQP surface layer, as stated previously. The ratio of GO–GRGDS:GO–GRGDS–AQP in the composite GO‐based membrane was optimized to 8:2 for realizing the lower usage of RBCM vesicles and higher water permeance and retaining the salt‐rejection capability. To determine the appropriate ratio, we tested various relative ratios of the two layers: GO–GRGDS:GO–GRGDS–AQP = 8:2, 6:4, and 0:10 (all GO–GRGDS–AQPs). Figure S7, Supporting Information presents the water permeance and NaCl rejection from a 1000‐ppm NaCl solution through these membranes. The water permeance and NaCl rejection is 8.84 LMH bar^−1^ with 89% NaCl rejection, 6.39 LMH∙bar^−1^ with 91.6%, and 6.17 LMH∙ bar^−1^ with 93% NaCl rejection for 8:2. 6:4 and 0:10 membranes, respectively. With an increasing relative amount of GO–GRGDS–AQP, the water permeance decreased and salt rejection increased, as was expected. As only the GO–GRGDS–AQP layer has an ion‐rejection capability (not GO–GRGDS as in GO–NPANPA), an increase in the relative amount of GO–GRGDS–AQP corresponds to an increase in the effective membrane thickness for filtration. As the 8:2 ratio resulted in a significantly higher water permeance with acceptable NaCl rejection capability, we have selected this ratio as a reference.

**Figure 4:**
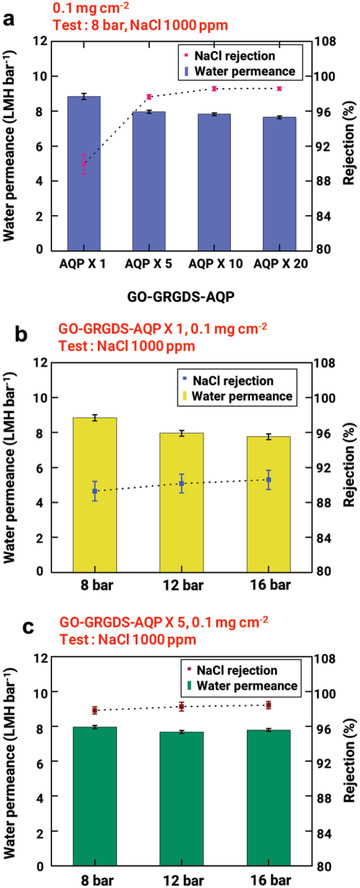
Effect of RBCM coverage of channels in GO membrane. a) Water permeance and NaCl rejection of GO–GRGDS–AQP(X 1), GO–GRGDS–AQP(X 5), GO–GRGDS–AQP(X 10), and GO–GRGDS–AQP(X 20). Pressure response of b) GO–GRGDS–AQP(X 1) and c) GO–GRGDS–AQP(X 5) membranes at 8, 12, and 16 bar.

Although the GO–GRGDS–AQP (8:2) membrane demonstrated a good water purification performance, the salt rejection of our GO–AQP membrane was still below the desired level (i.e., >99% rejection for NaCl). We speculate that this unsatisfactory salt rejection despite the incorporation of AQPs can be attributed to the insufficient coverage of the channels or defects in the GO membrane with RBCMs. As in our previous work,^[^ ^18^ ^]^ the interlayer space between the stacked GO layers (both in the plane and vertical directions) and defects in the basal plane of GO serve as channels for transport through the GO. Furthermore, the peptide‐functionalized GO membranes demonstrated a channel size of 1–2 nm, which is not sufficiently small to result in the rejection of salts such as NaCl and MgSO_4_. To achieve the desired salt‐rejection capability in our GO‐based membranes, the channels (defects and inter‐flake space) in the GO membranes should be blocked with RBCMs such that material transport can occur only through the AQPs. To fully cover the channels of GO with RBCM vesicles and thus improve the filtration properties of GO–GRGDS–AQP membranes for NaCl, the amount of RBCM vesicles mixed with the GO–GRGDS solution was adjusted to be 5, 10, and 20 times higher than the corresponding originally fabricated membranes. The fabricated membranes were denoted as GO–GRGDS–AQP (X 1), GO–GRGDS–AQP (X 5), GO–GRGDS–AQP (X 10), and GO–GRGDS–AQP (X 20), respectively. The transport properties were examined using a 1000‐ppm NaCl solution at an applied pressure of 8 bar, and the resulting water permeation and NaCl rejection are presented in **Figure** **4**a. GO–GRGDS–AQP (X 1) exhibited the greatest water permeance of 8.84 LMH bar^−1^, and its water permeance was slightly reduced with an amount of RBCM vesicles added to the GO–GRGDS solution; the water permeance of GO–GRGDS–AQP (X 5) and GO–GRGDS–AQP (X 10) was 7.96 LMH bar^−1^ and 7.83 LMH bar^−1^, respectively. However, as the amounts of RBCM increased from X 1 to X 5 and X 10, the NaCl rejection increased significantly from 89% to 98% and 99.1%, respectively. This result confirms the validity of our assumption that the channels in the GO membrane have not been sufficiently filled with RBCMs with the initial amount (X 1) and suitable coverage of channels in the GO membrane with RBCMs can block the passage of NaCl salt at the desired level (rejection >99%). However, the further increase of the RBCM addition to the GO–GRGDS solution up to 20 times higher than the initial value (GO–GRGDS–AQP (X 20)) resulted in saturation in both the water permeance and NaCl rejection. The water permeance and NaCl rejection of GO–GRGDS–AQP (X 20)) were similar to those of the GO–GRGDS–AQP (X 10) membrane. This indicates that the coverage of channels in GO membranes is saturated at approximately a 10‐times higher addition of RBCM vesicles to the GO–GRGDS solution. The interplanar distance of GO–GRGDS–AQP (X 5), estimated via XRD, was similar to that of GO–GRGDS–AQP (X 1) (Figure S8, Supporting Information). This indicates that an increased incorporation of RBCMs as compared to the initial value (X 1) does not increase the interplanar distance between the GO sheets. Therefore, the RBCMs do not stack vertically between themselves, but additional in‐plane coverage of channels in the GO membranes with RBCMs occurs instead with increased RBCM addition. We further assessed the pressure response of the GO–GRGDS–AQP membranes in verifying the mechanical integrity of the GO–GRGDS–AQP membranes. In typical polymeric membranes, the water permeance and pressure‐normalized flux remain almost constant unless the applied pressure reaches the maximum endurance point of the membrane. However, in peptide‐functionalized GO membranes, the water permeance of the membrane is inversely proportional to the applied pressure because the high applied pressure compresses the interlayer space between the GO sheets.^[^ ^18^ ^]^ The water permeance and NaCl rejection of the 1000‐ppm NaCl solution through the GO–GRGDS–AQP (X 1) membrane at various applied pressures are presented in Figure 4b. The water permeance of the GO–GRGDS–AQP (X 1) membrane decreased progressively with the increase in pressure (8.84, 7.96, and 7.84 LMH bar^−1^ at 8, 12, and 16 bar, respectively). In contrast, the GO–GRGDS–AQP (X 5) membrane behaved differently in response to the pressure increase (Figure 4c). The pressure‐normalized permeation flux hardly changed with the pressure increase (7.96, 7.68, and 7.79 LMH bar^−1^ at 8, 12, and 16 bar, respectively), which suggests that the applied pressure did not affect the interlayer space of the membrane. The NaCl rejection was also almost invariant (89, 90.1, and 90.7% at 8, 12, and 16 bar, respectively). Here, we found that the mechanical integrity of the GO–GRGDS–AQP membranes can be sufficiently increased when the channels in the GO membrane are sufficiently filled with RBCMs. Water filtration at a high pressure is critical for achieving significantly enhanced water flux; thus, our GO–GRGDS–AQP membranes appear to be promising as high‐pressure desalination membranes.

We examined the long‐term stability of the GO–GRGDS–AQP membranes with GO–GRGDS–AQP (X 5). The test was conducted using a 1000‐ppm NaCl solution at an applied pressure of 8 bar for 1 week, and the resulting water permeance and NaCl rejection are presented in Figure S9, Supporting Information. The membrane showed stable water permeance and NaCl rejection for 1 week. Since the physical stability of the RBCM for 4 weeks was verified in previous work,^[^ ^41^ ^]^ the integrity between GRGDS and RBCM was preserved for at least 1 week.

**Figure 5:**
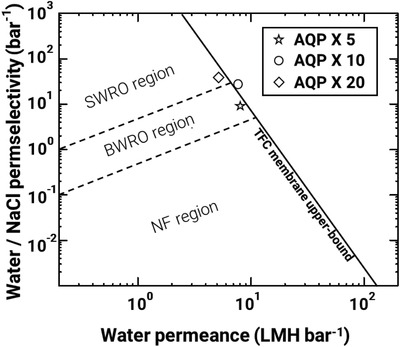
Performance of various GO‐GRGDS‐AQP desalination membranes. The upper‐bound line of commercial TFC membrane plotted as permselectivity versus water permeance.

The performance of the AQP‐implanted GO membrane developed in this study was compared with those of commercial polymeric membranes. Commercial high‐performance, ion‐rejecting water‐purification membranes are classified as seawater RO (SWRO) membranes, brackish RO (BWRO) membranes, and NF membranes. The majority of these membranes are based on TFC‐PA. Herein, our optimized GO–GRGDS–AQP membrane was tested under BWRO conditions (1000‐ppm NaCl solution, 8 ∼ 16 bar). In conventional membranes, an increase in the water permeance also increases the salt permeability, that is, the water permeance is inversely proportional to the salt rejection. **Figure** **5** presents the upper bound in the water‐permeance–NaCl‐rejection relationship for commercial TFC membranes. This upper‐bound line is plotted using water/NaCl permselectivity (A/B) versus water permeance (A). This upper‐bound line was previously presented in a study comprising the application of the solution‐diffusion model to commercial TFC membranes^[^ ^42^ ^]^ and was expressed via the equation: A/B = 16000A^−3.2^ (with A/B in bar^−1^ and A in LMH∙bar^−1^). The area below the upper‐bound line was divided into three regions based on the salt rejection property. The water permeance and salt rejection of commercial TFC BWRO membranes are limited by this upper‐bound line because of their permeability‐selectivity trade‐off. Our optimized GO–GRGDS–AQP (X 10) membrane surmounted the trade‐off upper bound of commercial TFC membranes. This superior desalination performance was facilitated by the reinforcement of the mechanical integrity of AQP‐incorporated GO membranes through molecular recognition between the cell‐binding ligand and integrin of the RBCMs. Although our optimized membrane surpasses the upper bound of commercial TFC membrane, the membrane showed NaCl rejection of 99.1% which is below the rejection performance of the advanced commercial RO membranes. With the deliberate control of the defects in the membrane, we expect further improvement in rejection performance of membrane developed in this study.

## 3 Conclusion

In conclusion, we demonstrated the successful transplantation of AQPs into the GO‐based membrane without the loss of its inherent function. The material‐specific binding between integrin of the RBCM and RGD ligand reinforced the mechanical strength of the GO–GRGDS–AQP membrane for enduring high‐pressure RO operation without performance degradation. RGD‐ligand‐mediated tethering of AQPs also ensured the desired movement of AQPs for their full function without frictional hindrance from the solid support. We also proved that solid‐state membranes with incorporated AQPs can exhibit superior AQP salt‐rejection properties even at high operating pressures of RO when the large channels/defects of the pristine solid‐state membrane are sufficiently covered/filled with biological membranes containing AQPs. In this study, a novel method of incorporating AQPs into GO membranes was developed, wherein RBCMs with AQPs were inserted between the GO flakes such that channels/defects in the GO‐based membranes were sufficiently covered with RBCMs. The developed GO–GRGDS–AQP membrane presented high water permeance and salt rejection simultaneously, thus surpassing the upper bound of the commercial TFC‐PA BWRO membrane in the water‐permeance–salt‐rejection relationship. The simple and scalable fabrication process of this membrane could pave the way for new opportunities in the application of high‐performance biomimetic desalination membranes.

## 4 Experimental Section/Methods

### Fabrication of GO‐Peptide (GO–GRGDS or GO–NPANPA)

GO was prepared using a modified Hummer's method,^[^ ^43^ ^]^ as reported previously. The aqueous GO solution was then further treated to increase the number of carboxylic functional groups on the GO surface: sodium chloroacetate (ClCH_2_COONa, 5 g) and sodium hydroxide (NaOH, 5 g) were added to the aqueous GO solution (100 mL) at a concentration of 1 mg∙mL^−1^ and bath‐sonicated in for 2 h. The resulting solution was neutralized with hydrochloric acid (HCl, 1 m) to form carboxylated GO (GO–COOH). Rinsing and centrifugation at 12 000 rpm for 15 min were repeated to purify the GO–COOH mixture. A phosphate buffer (pH 7.4, 100 mL) was then added to the final sediment to form a well‐dispersed GO–COOH solution. Carbodiimide coupling chemistry was applied to functionalize GO–COOH with peptides of interest (GRGDS or NPANPA, chemical structure of peptides is presented in Figure S10, Supporting Information): 1‐ethyl‐3‐(3‐dimethlyaminopropyl)‐carbodiimide (EDC, 20 mm) and *N*‐hydroxysuccinimide (NHS, 5 mm) were dissolved in a phosphate buffer (50 mL) and mixed with a GO–COOH solution (1 mg mL^−1^, 50 mL). The mixture was stirred for 10 min, bath‐sonicated for 1 h, and stirred for another 10 min. The resulting solution was rinsed and centrifuged several times at 1200 rpm for 10 min to remove the excess EDC and NHS. The final sediment was dissolved in the phosphate buffer (50 mL). This EDC–NHS‐activated GO–COOH solution was mixed with the peptide solution (GRGDS or NPANPA in phosphate buffer) and stirred overnight. Excess peptides and impurities were discarded by washing the solution several times with the phosphate buffer. The final sediment of the peptide‐functionalized GO (GO–GRGDS or GO–NPANPA) was dispersed in the phosphate buffer via bath sonication and tip sonication.

### Preparation of AQP‐Rich RBCM

RBCs were extracted from single donor human whole blood (EDTA K2 treated, Innovated Research, MI, USA). The following steps were conducted at 4 °C. The RBCs were isolated from the whole blood by centrifugation at 1000 rpm for 10 min and then washed three times with 1 × PBS. For hemolysis, the RBCs were suspended in 0.25 × PBS for 30 min. Subsequently, the RBCM fragments coexisting with hemoglobin were purified by centrifugation at 14 000 rpm for 30 min to eliminate the hemoglobin. The light pink sediment of the RBCM concentrate was collected and stored at −80 °C until used. For the fluorescence imaging, 5% carboxyfluorescein‐labeled phospholipid was added to the RBCM, and subsequently, the phospholipid was intercalated into the RBCM via sonication.

### Functionalization of RBCM on GO‐Peptide

The prepared RBCM sediment was diluted with PBS (3 mL) and bath‐sonicated for 5 min to obtain a well‐dispersed solution. The resulting solution was mixed with a GO‐based solution and bath‐sonicated for 10 min to functionalize the GO with RBCMs. During the sonication, the temperature of the solution was maintained below 30 °C using an ice pack. After the RBCM functionalization, the solution was centrifuged at 4000 rpm for 15 min to eliminate the excess RBCM vesicles. The final sediment was dispersed in a phosphate buffer via bath sonication. The resulting GO‐based material functionalized with GRGDS and RBCM was and named as GO–GRGDS–AQP, and the final concentration of the GO–GRGDS–AQP solution was adjusted to 0.1 mg mL^−1^ of GO–GRGDS–AQP.

### Characterization

GO‐based membranes were examined using XPS (K‐alpha+, Thermo Scientific, Waltham, MA, USA). RBCM‐ (and thus, AQP‐) incorporated GO‐based (GO–GRGDS–AQP or GO–NPANPA–AQP) membranes tagged with fluorescence markers were characterized using confocal microscopy (Nikon C1 PLUS, Minato, Tokyo, Japan). The diameter of the RBCM vesicles was measured using a dynamic light scattering (DLS, Malvern Panalytical Zetasizer Nano S90, Malvern, UK). The AQP‐incorporated GO membrane and peptide‐functionalized GO membranes were characterized via XRD using a D8 ADVANCE (Bruker, Billerica, MA, USA). Surface roughness of the membrane was measured by atomic force microscopy (AFM) using NX10 (Park systems, Suwon, South Korea). Cross‐sectional membrane images were characterized by scanning electron microscopy (SEM) using Nova NanoSEM 450 (FEI company, Hillsboro, OR, USA)

### Permeation Test

The water permeation and salt rejection were assessed in a stirred cell (Sterlitech HP4750 Stirred cell, Kent, WA, USA). The GO‐based membrane was fixed at the bottom of the stirred cell with the active area of 14.6 cm^2^. The stirred cell was filled with 300 mL of aqueous NaCl or MgSO_4_ solution. The stir‐rate for the stirred cell was set as 60 rpm. Pressure was applied using nitrogen gas from 8 to 16 bar. The solution permeated through the membrane was collected in a small plastic vessel. The volume of permeated solution was measured by weighing the solution at every 12 h. The test was conducted at least 3 days and all tests were at least triplicated for reproducibility.

### Evaluation of the Permeance and Salt Rejection

The solution used for the test was 1000 ppm of aqueous NaCl or MgSO_4_ solution at pH 7.4 ± 0.1. Prior to each permeation test, the membrane was compacted for at least 2 h at an applied pressure *P* = 8, 12, and 16 bar for each test, respectively. The flux, *J_w_*, of the membrane was calculated by dividing volume of permeated solution by the active are of the membrane and measured time. The permeance, *A*, was calculated by dividing *J_w_* by the applied pressure, $A=J_{w}/P$. The solute rejection, *R*, was calculated from the concentration of feed (*C_f_*) and permeated solution (*C_p_*), both obtained from electric conductivity measured with a conductivity meter S47‐k (Mettler‐Toledo, Columbus, OH, USA); $R=1-C_{p}/C_{f}$.

## Conflict of Interest

The authors declare no conflict of interest.

## Supporting information

Supporting Information

## Data Availability

Research data are not shared.
