# Supplementary material for: Aquaporin‐Incorporated Graphene‐Oxide Membrane for Pressurized Desalination with Superior Integrity Enabled by Molecular Recognition
Source: Adv Sci (Weinh). 2021 Aug 16;8(20):2101882. doi: 10.1002/advs.202101882 (PMC8529452; doi:10.1002/advs.202101882)
Supplement: Supplementary file 1 — Supporting Information [file ADVS-8-2101882-s001.pdf]

© 2021 Wiley-VCH GmbH

ADVANCED SCIENCE
Open Access

Supporting Information

for *Adv. Sci.*, DOI: 10.1002/advs.202101882

Aquaporin-Incorporated Graphene-Oxide Membrane for Pressurized Desalination with Superior Integrity Enabled by Molecular Recognition

Chang Seon Lee, Insu Kim, Dae sung Yoon and Yun Jung Lee**

© 2020 Wiley-VCH GmbH

Supplementary Information

Aquaporin-Incorporated Graphene-Oxide Membrane for Pressurized Desalination with Superior Integrity Enabled by Molecular Recognition

*Chang Seon Lee, Insu Kim, Dae sung Yoon*and Yun Jung Lee**

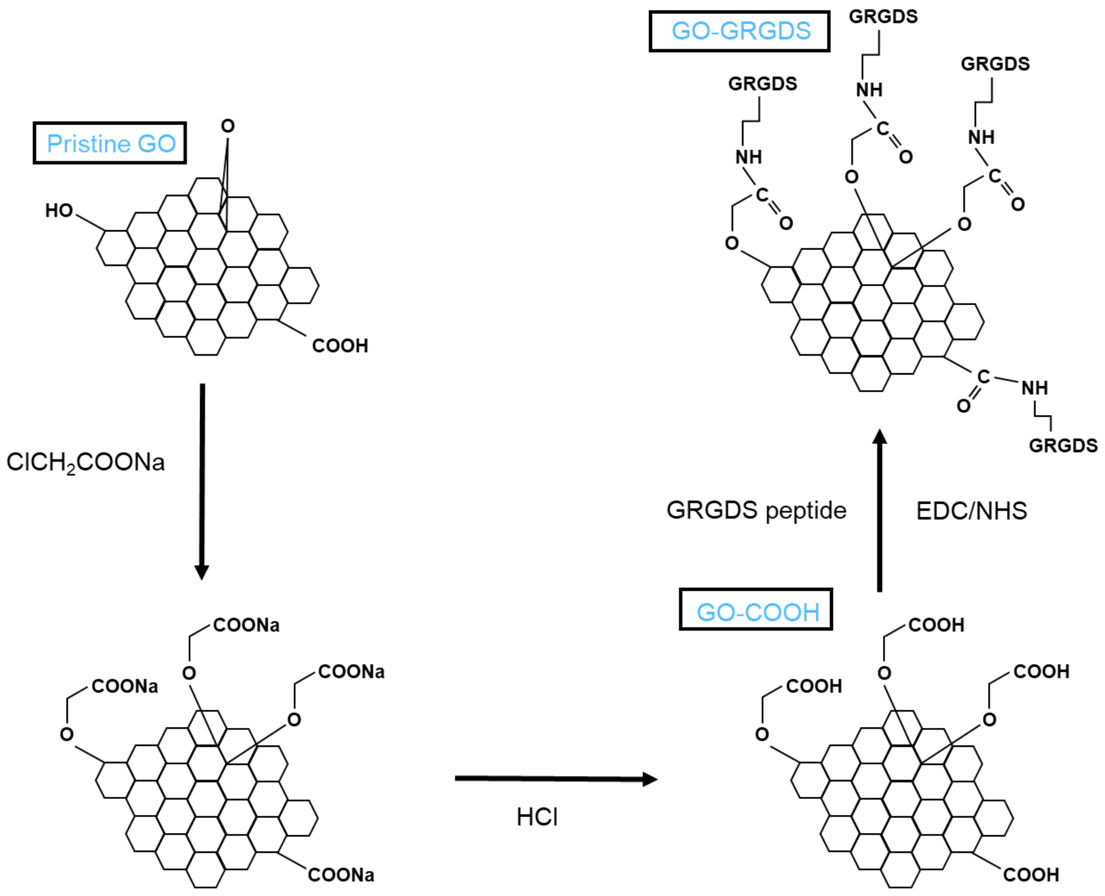

Figure S1. Schematic of procedure for GO–GRGDS functionalization.

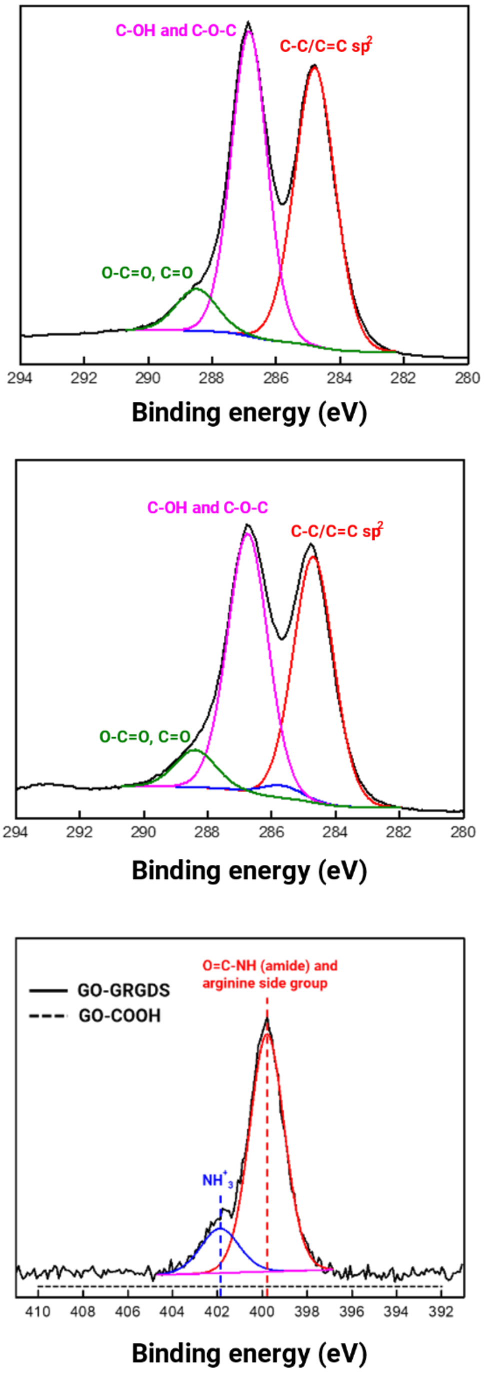

Figure S2. High-resolution XPS spectra of GO–COOH and GO–GRGDS. (a) C1s spectra of GO–COOH, (b) C1s spectra of GO–GRGDS, and (c) N1s spectra of GO–COOH and GO–GRGDS.

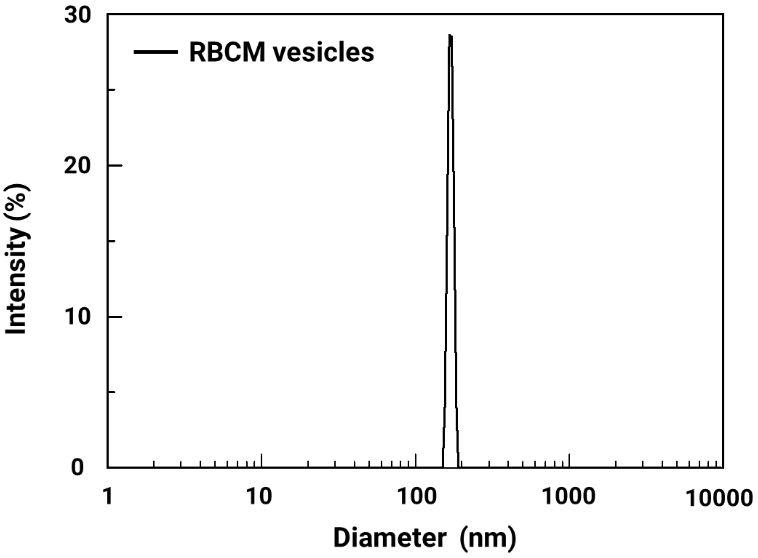

Figure S3. Diameter of RBCM vesicles measured using DLS.

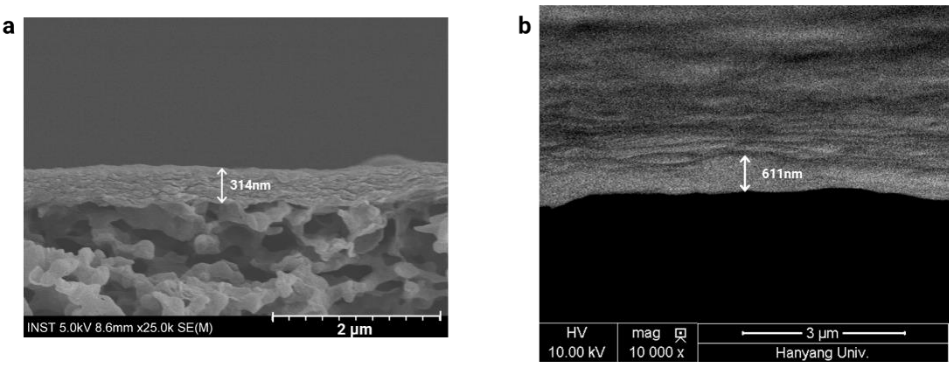

Figure S4. The cross-sectional SEM image of (a) GO-GRGDS, (b) GO-GRGDS-AQP.

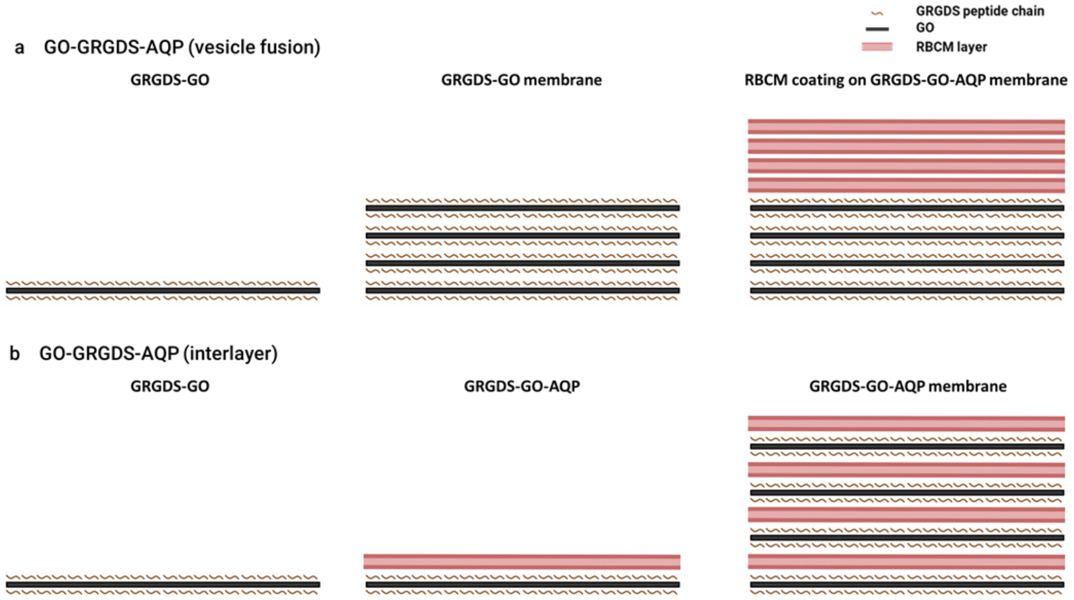

Figure S5. Schematics of RBCM vesicle incorporation into GO membranes via (a) vesicle fusion coating method and (b) interlayer method.

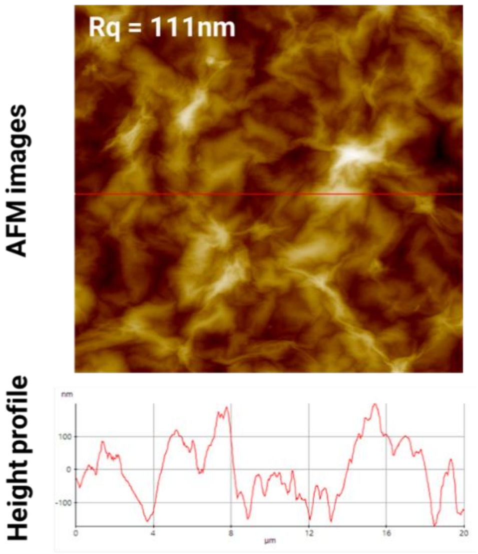

Figure S6. Height profile of GO–GRGDS measured using atomic force microscopy (AFM).

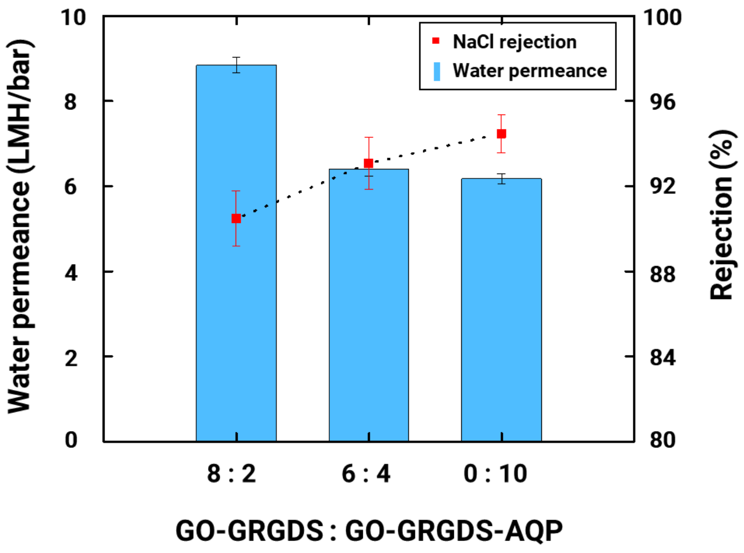

Figure S7. Performance of GO–GRGDS–AQP membrane fabricated with the GO–GRGDS/GO–GRGDS–AQP ratio of 8:2, 6:4, and 0:10.

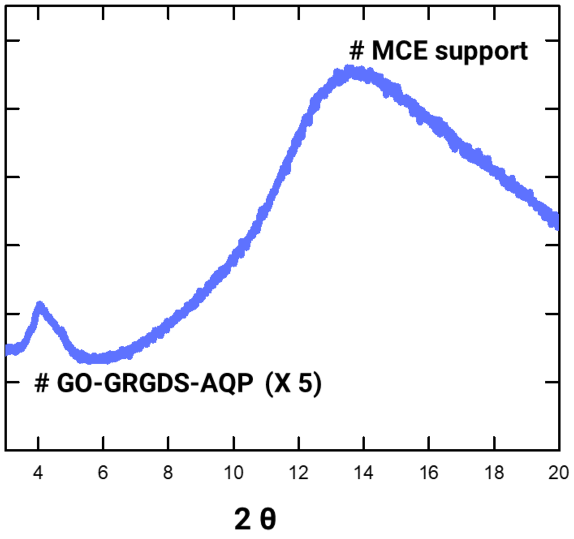

Figure S8. XRD of GO–GRGDS–AQP (X 5) membrane.

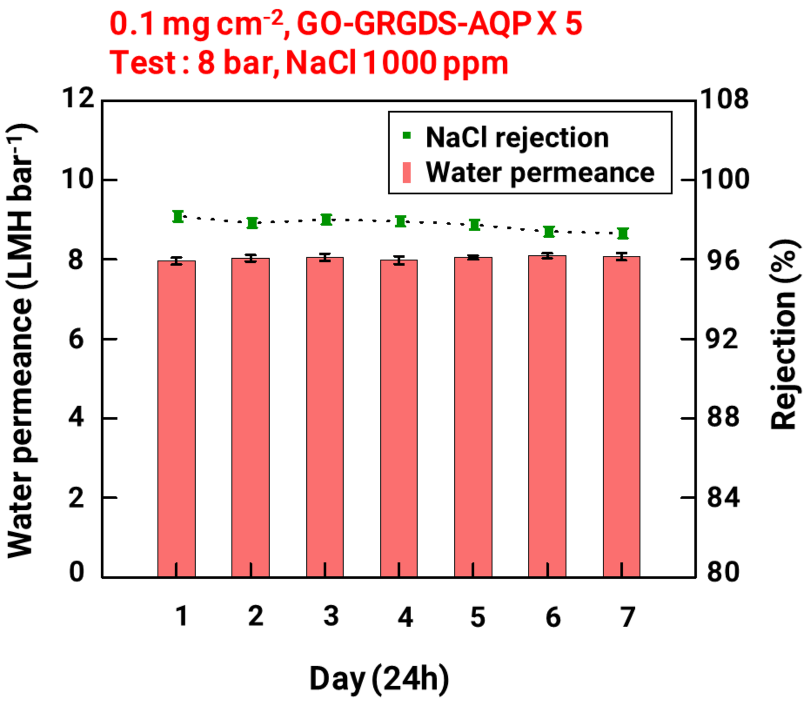

Figure S9. Performance retention of GO-GRGDS-AQP X 5 for 7 days.

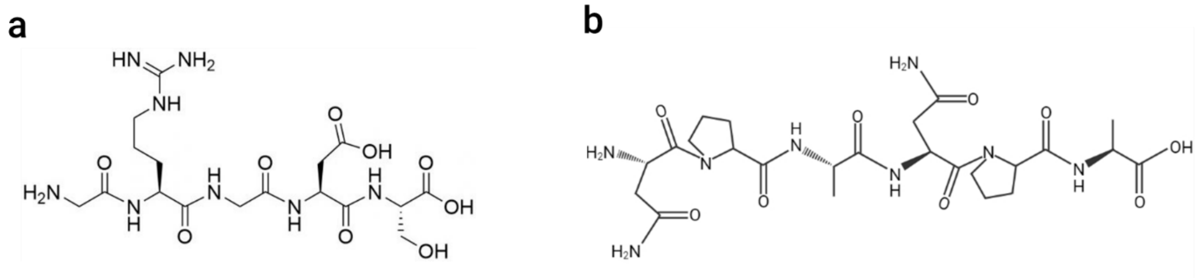

Figure S10. Chemical structure of peptides. (a) GRGDS, (b) NPANPA.
